# Supplementary material for: Similar excitability through different sodium channels and implications for the analgesic efficacy of selective drugs
Source: eLife. 2024 Apr 30;12:RP90960. doi: 10.7554/eLife.90960 (PMC11060714; doi:10.7554/eLife.90960)
Supplement: Supplementary file 1. — A, Model data before and after channel “inhibition”. B, Reagents. C, EC50 values. D, Primers. E, Model equations. F, Conductance densities at baseline for DIV 0 and DIV4-7 models. [file elife-90960-supp1.docx]

*SUPPLEMENTARY FILE 1*

*Table A. Model data before and after channel “inhibition”*

|  | **DIV0** | | **DIV4-7** | | |
| --- | --- | --- | --- | --- | --- |
|  | Baseline | Na_V_1.8 | Baseline | Na_V_1.7 | Na_V_1.3 |
| **Rheobase (pA)** | 17 | 22 | 12 | 16 | 21 |
| **Spike height (mV)** | 72.8 | 46.9 | 57.1 | 21.4 | 52 |
| **RMP (mV)** | -69.0 | -69.7 | -70.0 | -69.8 | -70.4 |

***Table B. Reagents***

| Reagent | Description | Source |
| --- | --- | --- |
| TTX | Tetrodotoxin citrate | Alomone |
| PF-24 | PF-01247324 | Sigma |
| PF-71 | PF-05089771 | Alomone |
| ICA | ICA-121431 | Tocris |
| Papain | Papain lyophilized power | Worthington |
| Collagenase | Collagenase type II | Worthington |
| Dispase II | Dispase type II | Sigma |

***Table C. EC50 values***

| Drug (concentration used) | **EC50 values** | | | **References** |
| --- | --- | --- | --- | --- |
|  | **Na_V_1.3** | **Na_V_1.7** | **Na_V_1.8** |  |
| TTX (100 nM) | **4 nM** | **2 nM** | 46±10 μM | (21, 96) |
| ICA-121431 (1 μM) | **20 nM** | >10 μM | >10 μM | (53) |
| PF05089771 (30 nM) | 11 μM | **11 nM** | >10 μM | (40) |
| PF-01247324 (1 μM) | >30 μM | >30 μM | **331 nM** | (50) |
| A-803467 (1 μM) | 2.5 μM | 6.7 μM | **8 nM** | (97) |

Bolded entries indicate the Na_V_ subtype that is selectively blocked

***Table D. Primers***

|  | HPRT | Na_V_1.7 | Na_V_1.8 |
| --- | --- | --- | --- |
| NCBI Reference Sequence | *NM_013556.2* | *NM_001290674.1* | *NM_001205321.1* |
| Length | 144 | 114 | 148 |
| Forward | TCCTCCTCAGACCGCTTT | TGATGGTCATGGTGATTGGG | AGCCATCAAAGTGTCCGTC |
| Reverse | TTTTCCAAATCCTCGGCATAATG | TGTTTGCGTCGGTGTCTTC | CTTTATCAGAGCCTCGAAGGTG |

*Table E. Model equations*

| **Channel Model** | **Equations** | |
| --- | --- | --- |
| Na_V_1.7 (86) | $I_{NaV1.7}=\bar{g}_{NaV1.7}m^{3}h(V-E_{Na})$ | |
|  | $\dot{m}=\alpha_{m}(1-m)-m\beta_{m}$ | $\dot{h}=\alpha_{h}(1-h)-h\beta_{h}$ |
|  | $\alpha_{m}=10.22-\frac{10.22}{1+\exp\left( \frac{V+7.19}{15.43} \right)}$ | $\alpha_{h}=\frac{0.0744}{1+exp\left( \frac{V+99.76}{11.07} \right)}$ |
|  | $\beta_{m}=\frac{23.76}{1+\exp\left( \frac{V+70.37}{14.53} \right)}$ | $\beta_{h}=2.54-\frac{2.54}{1+exp\left( \frac{V+7.8}{10.68} \right)}$ |
| Na_V_1.3* | $I_{NaV1.3}=\bar{g}_{NaV1.3}m^{3}h(V-E_{Na})$ | |
|  | $\dot{m}=\alpha_{m}(1-m)-m\beta_{m}$ | $\dot{h}=\alpha_{h}(1-h)-h\beta_{h}$ |
|  | $\alpha_{m}=10.22-\frac{10.22}{1+\exp\left( \frac{V+7.19+12}{15.43} \right)}$ | $\alpha_{h}=\frac{0.0744}{1+exp\left( \frac{V+99.76}{11.07} \right)}$ |
|  | $\beta_{m}=\frac{23.76}{1+\exp\left( \frac{V+70.37+12}{14.53} \right)}$ | $\beta_{h}=2.54-\frac{2.54}{1+exp\left( \frac{V+7.8}{10.68} \right)}$ |
| Na_V_1.8 (87) | $I_{NaV1.8}=\bar{g}_{NaV1.8}m^{3}h(V-E_{Na})$ | |
|  | $\dot{m}=\alpha_{m}(1-m)-m\beta_{m}$ | $\dot{h}=\alpha_{h}(1-h)-h\beta_{h}$ |
|  | $\alpha_{m}=7.21-\frac{7.21}{1+\exp\left( \frac{V-0.063}{7.86} \right)}$ | $\alpha_{h}=0.003+\frac{1.63}{1+exp\left( \frac{V+68.5}{10.01} \right)}$ |
|  | $\beta_{m}=\frac{7.4}{1+\exp\left( \frac{V+53.06}{19.34} \right)}$ | $\beta_{h}=0.81-\frac{0.81}{1+exp\left( \frac{V-11.44}{13.12} \right)}$ |
| K_M_ (88) | $I_{KM}=\bar{g}_{KM}n(V-E_{K})$ | |
|  | $\dot{n}=\frac{n_{\infty}-n}{\tau_{n}}$ | |
|  | $n_{\infty}=\frac{1000}{1+e^{-\frac{V+35}{5}}}$ | $\tau_{n}=\frac{1000}{3.3e^{V+\frac{35}{10}}+e^{-\frac{V+35}{10}}}$ |
| K_dr_ (88) | $I_{KA}=\bar{g}_{KA}n^{3}l(V-E_{K})$ | |
|  | $\dot{n}=\alpha_{m}(1-m)-m\beta_{m}$ | $\dot{l}=\alpha_{h}(1-h)-h\beta_{h}$ |
|  | $\alpha_{n}=\frac{e^{(-5\times{10}^{-3}\cdot\left( V+32 \right)\cdot9.648\times{10}^{4})}}{2562.35}$ | $\alpha_{l}=\frac{e^{\left( 2\times{10}^{-3}\cdot\left( V+61 \right)\cdot9.648\times{10}^{4} \right)}}{2562.35}$ |
|  | $\beta_{n}=\frac{e^{(-2\times{10}^{-3}\cdot\left( V+32 \right)\cdot9.648\times{10}^{4})}}{2562.35}$ | $\beta_{l}=\frac{e^{(-2\times{10}^{-3}\cdot\left( V+32 \right)\cdot9.648\times{10}^{4})}}{2562.35}$ |
| AHP** | $I_{AHP}=\bar{g}_{AHP}z(V-E_{K})$ | $\dot{z}=\frac{1}{1+e^{5-V/4}}-\frac{z}{100}$ |

* Modified from Na_V_1.7: a hyperpolarizing shift of 12mV in V1/2 of activation gate, m

** Modified from (49)

Note that the Na_V_1.7 and Na_V_1.8 equations above do not include the liquid junction potential correction of 4.2 mV (86) and 5.3 mV (87), respectively, that were applied in the neuron model.

*Table F. Conductance densities at baseline for DIV 0 and 4-7 models*

|  | **Conductance densities at baseline (mS/cm^2^)** | | | | | | |
| --- | --- | --- | --- | --- | --- | --- | --- |
|  | ${\bar{\boldsymbol{g}}}_{\mathbf{Nav1.3}}$ | ${\bar{\boldsymbol{g}}}_{\mathbf{Nav1.7}}$ | ${\bar{\boldsymbol{g}}}_{\mathbf{Nav1.8}}$ | ${\bar{\boldsymbol{g}}}_{\mathbf{Kdr}}$ | ${\bar{\boldsymbol{g}}}_{\mathbf{M}}$ | ${\bar{\boldsymbol{g}}}_{\mathbf{AHP}}$ | ${\bar{\boldsymbol{g}}}_{\mathbf{Leak}}$ |
| **DIV 0** | 0 | 3 | 30 | 3 | 0.05 | 3.5 | 0.025 |
| **DIV 7** | 0.35 | 35 | 0.2 | 3.5 | 0.5 | 2.5 | 0.035 |
