# Supplementary material for: Similar excitability through different sodium channels and implications for the analgesic efficacy of selective drugs
Source: eLife. 2024 Apr 30;12:RP90960. doi: 10.7554/eLife.90960 (PMC11060714; doi:10.7554/eLife.90960)
Supplement: Source code 1. [file elife-90960-code1.zip › DRG_paper_code/readme.rtf]

Files: 1) smallDRG_DIV0.m for DIV0 simulations2) smallDRG_DIV7.m for DIV4-7 simulationsTo get started,1) Type smallDRG_DIV0(17,1500,500,1000) in the command line to reproduce DIV0 baseline. 2) Type smallDRG_DIV7(12,1500,500,1000) in the command line to reproduce DIV4-7 baseline. To reproduce figures: 3) Uncomment line 42 in smallDRG_DIV0.m to simulate Nav1.8 blockade in Fig.2D.4) Uncomment line 43 and 44, respectively, in smallDRG_DIV7.m to simulate Nav1.7 or 1.3 blockade in Fig.3D.5) Uncomment line 45 in smallDRG_DIV0.m for virtual Nav1.7 or line 47 in smallDRG_DIV7.m for virtual Nav1.8 in Fig.4A.6) Uncomment line 51 or 52 in smallDRG_DIV7.m to reproduce Fig.S4.
